# Heterogeneous frailty trajectories and their differential impact on social integration in older adults with COPD: a prospective longitudinal study

**DOI:** 10.3389/fpubh.2026.1824141

**Published:** 2026-05-08

**Authors:** Li Feng, Haiyan Ji, Zheng Jiang, Mengyao Liang

**Affiliations:** 1 Department of Nursing, The Sixth People's Hospital of Nantong, Nantong, Jiangsu, China; 2 Department of Respiratory Medicine, The Sixth People's Hospital of Nantong, Nantong, Jiangsu, China; 3 Department of Critical Care Medicine, The Sixth People's Hospital of Nantong, Nantong, Jiangsu, China

**Keywords:** chronic obstructive pulmonary disease, frailty, older adults, prospective studies, social integration, trajectory analysis

## Abstract

**Objective:**

To explore the heterogeneous development trajectories of frailty in older adults with chronic obstructive pulmonary disease (COPD) and to examine the associations between different trajectory classes and levels of social integration at 12 months.

**Methods:**

A prospective longitudinal study was conducted. Eligible older adults with COPD were consecutively recruited from the Sixth People’s Hospital of Nantong City between May 2024 and January 2025. Assessments were performed at five time points: baseline (T0), 3 months (T1), 6 months (T2), 9 months (T3), and 12 months (T4). Frailty was assessed using the Fried frailty phenotype. Social integration was evaluated across three dimensions: functional, structural, and subjective experience, using the Instrumental Activities of Daily Living scale, the 6-item Lubben Social Network Scale, and the UCLA Loneliness Scale (Version 3), respectively. Data analysis involved identifying potential frailty development trajectory classes using growth mixture modeling. Hierarchical multiple linear regression models were employed to examine the independent associations between different frailty trajectory classes and 12-month social integration outcomes, while controlling for baseline social integration levels, demographic, and clinical variables.

**Results:**

A total of 347 patients completed all five follow-ups. Growth mixture modeling identified four distinct frailty development trajectories, in order of class proportion: Persistently Stable-Low Frailty (41.2%), Slow Progression (33.1%), Post-Exacerbation Fluctuation (18.5%), and Rapid Deterioration (7.2%). The four trajectory groups differed significantly in baseline characteristics such as age, lung function, and number of acute exacerbations in the past year (all *p* < 0.05). At the 12-month follow-up, significant gradient differences were observed among the four groups in scores and change scores from baseline across all three social integration dimensions (all *p* < 0.001), with the Rapid Deterioration group having the worst outcomes in all dimensions. Hierarchical multiple linear regression analysis showed that, after controlling for relevant confounding factors, the Rapid Deterioration trajectory was independently associated with worse instrumental activities of daily living, smaller social network size, and greater loneliness compared to the Persistently Stable-Low Frailty group (all *p* < 0.05). The strength of these associations exhibited a gradient increase from the Slow Progression to the Rapid Deterioration trajectory.

**Conclusion:**

Frailty development in older adults with COPD follows four heterogeneous trajectories, among which the Rapid Deterioration and Post-Exacerbation Fluctuation trajectories hold important clinical warning significance. Different frailty trajectory classes are independently associated with long-term social integration outcomes. In clinical practice, dynamically assessing frailty trajectories and stratifying patients based on risk has potential value for implementing targeted interventions to maintain their social functioning.

## 1 Introduction

Chronic obstructive pulmonary disease (COPD) is a leading global cause of disability and mortality, with its disease burden becoming increasingly significant in aging societies (1). Older adults with COPD face not only the challenge of progressive respiratory decline but also a high prevalence of geriatric syndromes, among which frailty is particularly prominent (2). Studies indicate that the risk of developing frailty in COPD patients is more than twice that of their healthy peers, with prevalence rates ranging from 9 to 28% (3). Frailty, defined as a dynamic, multidimensional state of decreased physiological reserve, significantly increases the risks of acute exacerbations, hospitalization, disability, and mortality, constituting a core difficulty in disease management (4–6).

However, existing evidence has two key limitations. First, from a cognitive perspective (6, 7), current research is largely based on the assumption of “population homogeneity.” While numerous cross-sectional studies have confirmed a strong association between COPD and frailty, the few longitudinal studies focusing on transitions in frailty status generally treat patients as a homogeneous group, depicting an “average” change trend. This overlooks the clinically evident reality that frailty progression can vary drastically among different patients—some declining rapidly, others remaining stable long-term, and yet others experiencing recurrent fluctuations. The existence of these heterogeneous trajectories, their underlying driving factors, and their clinical significance have not been systematically explored. Second, regarding outcome focus (8, 9), there is a tendency in the literature to prioritize “physiological over social” endpoints. Existing studies widely discuss the impact of frailty on hard endpoints like mortality and rehospitalization in COPD patients but relatively neglect its profound effect on the soft endpoint of psychosocial function. Social integration (10), defined as an individual’s ability to maintain social roles, participate in social activities, and perceive a sense of belonging, is a core dimension of healthy aging. For COPD patients, dyspnea, activity limitation, and disease-related stigma easily lead to social withdrawal and isolation, and frailty may exacerbate this vicious cycle (11, 12). Unfortunately, how frailty dynamically influences social integration remains poorly understood. Theoretically, the Fear-Avoidance Model provides a robust explanatory framework linking frailty and social integration (13, 14). Patients may develop fear due to dyspnea, subsequently avoiding physical and social activities. This leads to further physical deconditioning and shrinkage of social networks, ultimately trapping them in a vicious cycle of “dyspnea-fear-avoidance-frailty-social isolation.” The varying speed and depth with which different patients become “trapped” in this cycle may be a key psychobehavioral mechanism underlying the heterogeneity of their frailty trajectories.

Therefore, overcoming the limitations of cross-sectional designs by employing a prospective longitudinal study to dynamically delineate the heterogeneous development trajectories of frailty in older adults with COPD and empirically test their differential impacts on social integration holds significant scientific and practical importance. This study aims to: (1) identify potential heterogeneous frailty trajectory classes in older adults with COPD using Growth Mixture Modeling; (2) compare the baseline characteristics and endpoint social integration levels of patients across different trajectory classes. The findings will provide key evidence for shifting the nursing paradigm from a “one-size-fits-all” approach toward “precision intervention based on trajectory prediction,” thereby contributing to the improvement of overall health outcomes and quality of life for older adults with COPD.

## 2 Participants and methods

### 2.1 Study participants

This prospective longitudinal cohort study was designed to dynamically capture frailty trajectories over 12 months, with assessments at five timepoints: baseline (T0), 3 months (T1), 6 months (T2), 9 months (T3), and 12 months (T4). To ensure complete 12-month follow-up data, eligible older adults with COPD were consecutively recruited from five inpatient wards and specialist outpatient clinics within the Department of Respiratory and Critical Care Medicine at the Sixth People’s Hospital of Nantong City between May 1, 2024, and January 31, 2025. (1) Inclusion criteria: ① Age ≥ 60 years; ② Meet the diagnostic criteria of the Global Strategy for the Diagnosis, Management, and Prevention of Chronic Obstructive Pulmonary Disease (2024 report) (15), i.e., post-bronchodilator forced expiratory volume in 1 s/forced vital capacity (FEV₁/FVC) < 0.70, and clinical features consistent with COPD; ③ Be in a clinically stable phase (no acute exacerbation for at least 4 weeks prior to screening); ④ Be conscious and possess basic communication and comprehension abilities; ⑤ Provide informed consent voluntarily and sign the informed consent form. (2) Exclusion criteria: ① Co-existing other severe respiratory diseases that may dominate the clinical presentation; ② Co-existing severe cognitive impairment, aphasia, mental illness, or terminal illness with an expected survival of <12 months or inability to cooperate with the study; ③ Having severe visual or hearing impairment affecting questionnaire assessment; ④ Planning to relocate from the local area within the next 12 months or explicitly stating inability to complete follow-up.

The core analysis of this study utilized Growth Mixture Modeling (GMM) to identify heterogeneous trajectories. According to methodological literature on GMM and sample size calculation guidelines (16), the minimum sample size required for model fitting is typically at least 50–100 cases per potential class. Literature suggests (17) that frailty trajectories in COPD patients may exhibit 3–4 potential classes. To ensure statistical power and with reference to similar published longitudinal trajectory studies, a medium effect size (Cohen’s *f* = 0.20) was preset. Power analysis for one-way ANOVA (used to compare differences in endpoint social integration among different trajectory groups) was performed using G*Power 3.1 software, setting *α* = 0.05, 1-*β* = 0.80, and number of groups = 4, resulting in a required total sample size of approximately 280. Considering the high risk of loss to follow-up in a 12-month longitudinal study with 5 time points, an attrition rate of 25% was anticipated. Therefore, the required initial sample size was calculated as: *N* = 280/(1–0.25) ≈ 373. In summary, this study planned to recruit at least 373 patients. This study was approved by the Ethics Committee of the Sixth People’s Hospital of Nantong City (Approval No.: NTLYLL2024007), and all patients provided informed consent.

### 2.2 Study instruments

#### 2.2.1 General information questionnaire

A self-designed questionnaire was used to collect information on patient age, gender, years of education, marital status, living arrangements, smoking history, and disease duration. Clinical data, including COPD GOLD grade, COPD Assessment Test (CAT) score, number of acute exacerbations in the past year, and number of comorbidities, were extracted from the hospital medical record system.

#### 2.2.2 Frailty assessment

The internationally recognized Fried frailty phenotype was used to objectively and multidimensionally assess the frailty status of patients. Proposed by Fried et al. in 2001 (18) and based on the “frailty phenotype” conceptual model, this scale identifies frailty through five physiological indicators and has good predictive validity. Judgment was based on the grip strength cut-off values recommended by the Asian Working Group for Sarcopenia for this study (19): male < 28 kg, female < 18 kg. Falling below the cut-off scores 1 point. ④ Slow Walking Speed: The patient’s usual gait speed was measured. A 4-meter line was marked on level ground, and the time (seconds) taken to walk the middle 3 meters at usual pace was recorded to calculate speed (m/s). Based on Fried’s original criteria and referencing data from Chinese populations (20), the following cut-offs were used: for males with height ≤ 173 cm and all females, speed ≤ 0.8 m/s; for males with height > 173 cm, speed ≤ 0.65 m/s. Falling below the cut-off scores 1 point. ⑤ Low Physical Activity: The short form of the International Physical Activity Questionnaire was used to assess overall physical activity level in the past week, converted to metabolic equivalents. According to Fried’s criteria, weekly activity expenditure < 383 kcal for males and <270 kcal for females scores 1 point. The number of criteria met is summed to obtain a frailty score (range 0–5). Classification follows international standards: non-frail: 0 criteria (score 0); pre-frail: 1–2 criteria (score 1–2); frail: ≥3 criteria (score 3–5).

#### 2.2.3 Social integration assessment

Social integration is a multidimensional concept referring to an individual’s participation, connection, and sense of belonging within social relationship networks. To comprehensively and objectively assess the social integration level of older adults with COPD, this study conducted a comprehensive measurement across three core dimensions: “functional integration,” “structural integration,” and “subjective integration experience,” using specific, widely validated scales for each dimension.

##### 2.2.3.1 Functional integration: assessment of instrumental activities of daily living

To assess the advanced self-care abilities essential for maintaining social roles, i.e., the level of functional integration, in older adults with COPD, this study used the widely employed Instrumental Activities of Daily Living (IADL) scale. This scale, derived from the Activities of Daily Living scale developed by Lawton and Brody (21), is specifically designed to measure the complex skills required for independent community living. Each item is rated on a 4-point scale, from “1 point” (needs no help, can do independently) to “4 points” (completely unable to do, completely dependent on others). The total score ranges from 8 to 32 points. A higher score indicates a greater degree of functional dependence and poorer functional social integration. In the sample data collected for this study, the scale demonstrated good internal consistency reliability, with a Cronbach’s *α* coefficient of 0.816, indicating it is a stable and reliable measurement tool.

##### 2.2.3.2 Structural integration: assessment of social networks

To objectively assess the social network resources of older adults with COPD, i.e., the level of structural integration, this study employed the Lubben Social Network Scale (LSNS-6) (22). This scale is specifically designed to assess the support potential of family and friend networks in older adults and is an effective tool for identifying the risk of social isolation. The LSNS-6 consists of 6 items, each item is rated on a 6-point scale from 0 to 5. The total score ranges from 0 to 30. A higher score indicates a larger social network size, closer connections, and better structural integration. According to the commonly used cutoff, a total score below 12 suggests a risk of social isolation, while a subscale score (family or friend) below 6 suggests isolation tendency within the respective network. The scale is concise, efficient, widely used in older populations, and has good reliability and validity. In the sample of this study, it showed ideal internal consistency reliability, with a Cronbach’s *α* coefficient of 0.809, indicating reliable measurement results.

##### 2.2.3.3 Subjective integration experience: assessment of perceived loneliness

To assess the subjective feelings of older adults with COPD regarding the quality of their social relationships, i.e., their subjective integration experience, this study used the internationally recognized UCLA Loneliness Scale (Version 3) (23). This version contains 20 items, each item is rated on a 4-point Likert scale describing the frequency of the feeling: 1 point (never), 2 points (rarely), 3 points (sometimes), 4 points (often). Reverse items require recoding before scoring. Scoring and Interpretation: The scores of all items are summed to obtain a total score ranging from 20 to 80. A higher total score indicates stronger perceived loneliness and poorer subjective social integration experience. Based on widely applied clinical cutoffs, a total score of 20–34 is generally considered “low loneliness,” 35–43 as “moderate loneliness,” and a score of 44 or above suggests “significant, high-level loneliness,” carrying clear clinical significance. In the sample of this study, the scale showed extremely high internal consistency reliability, with a Cronbach’s *α* coefficient of 0.909, indicating stable and reliable measurement results.

### 2.3 Data collection methods and quality control

This study was a prospective longitudinal study with a total of five assessment time points: baseline, 3 months, 6 months, 9 months, and 12 months after enrollment. Data collection was conducted through a combination of face-to-face interviews, standardized physical measurements, and extraction of medical record data. At baseline and each follow-up time point, trained and certified research staff interviewed patients in the outpatient clinic. Structured questionnaires were used to collect multidimensional data on sociodemographics, psychosocial and behavioral factors, and social integration. Calibrated tools, such as dynamometers and stopwatches, were used to measure objective indicators like grip strength and walking speed following standardized operating procedures. Clinical data, including disease diagnosis, pulmonary function, and acute exacerbation history, were extracted directly from the hospital’s electronic medical record system. To ensure the reliability of the study data, a series of rigorous quality control measures were implemented. All data collectors underwent standardized protocol training and passed a consistency assessment before the study began to ensure uniformity in assessment standards and operational procedures. A standardized follow-up procedure was established, coordinated by a dedicated person who maintained contact with patients via telephone, text messages, and other methods to minimize loss to follow-up. In data management, a dual independent entry system was used for inputting data into an electronic database, followed by consistency checks. Regular logic checks and data cleaning were also performed.

### 2.4 Statistical methods

All statistical analyses in this study were performed using R software (version 4.3.1). Continuous variables were assessed for normality using the Shapiro–Wilk test. Normally distributed data are presented as mean ± standard deviation, while non-normally distributed data are described as median (interquartile range). Categorical variables are reported as frequency and percentage. To identify heterogeneous frailty development trajectories, latent class growth analysis was conducted by fitting growth mixture models (GMM) using the mclustpackage. The model was specified as a linear unconditional growth model with the functional form: η_i = α_c + β_c * Time + ζ_i, where η_i represents the latent frailty score for individual *i* at a given time point, α_c and β_c denote the intercept (initial frailty level) and slope (rate of frailty change) for latent class c, respectively, and ζ_i is the individual-specific residual. Within each class, ζ_i was assumed to follow a multivariate normal distribution with an unstructured variance–covariance matrix (∑_c). Models with 1 to 5 latent classes were compared sequentially. The optimal number of classes was determined by comprehensively evaluating the Akaike Information Criterion (AIC), Bayesian Information Criterion (BIC), entropy (values > 0.8 preferred), and Bootstrapped Likelihood Ratio Test (BLRT) results, while ensuring class interpretability and a minimum class proportion greater than 5%.

After determining the trajectory classes, baseline characteristics were compared across groups using one-way analysis of variance (ANOVA), Kruskal–Wallis *H* test, or chi-square test, as appropriate. Social integration outcomes at 12 months and their change scores from baseline were similarly compared across trajectory groups. To assess the independent association of frailty trajectories with 12-month social integration outcomes, hierarchical multiple linear regression analysis was performed using the lmfunction. Three nested models were constructed: Model 1 adjusted for the baseline score of the corresponding outcome; Model 2 added demographic variables (age, sex, and years of education); Model 3 further included clinical variables (FEV₁% predicted, annual frequency of acute exacerbations). All models used the “Persistently Stable-Low Frailty” trajectory as the reference group. Exploratory and sensitivity analyses included: (1) sensitivity analysis of trajectory classification, involving refitting the GMM using data from the first 6 months and assessing the agreement with the 12-month model classification via Cohen’s Kappa coefficient calculated using the irrpackage; (2) interaction analysis, by adding interaction terms (“frailty trajectory × lung function group” [dichotomized by median FEV₁% predicted] and “frailty trajectory × acute exacerbation frequency group” [dichotomized by median annual exacerbation count]) to the final regression models. For longitudinal missing data, the GMM employed the full information maximum likelihood method for handling. All statistical tests were two-sided, with a *p*-value < 0.05 considered statistically significant.

## 3 Results

### 3.1 Participant enrollment and identification of heterogeneous frailty trajectories

**Figure 1:**
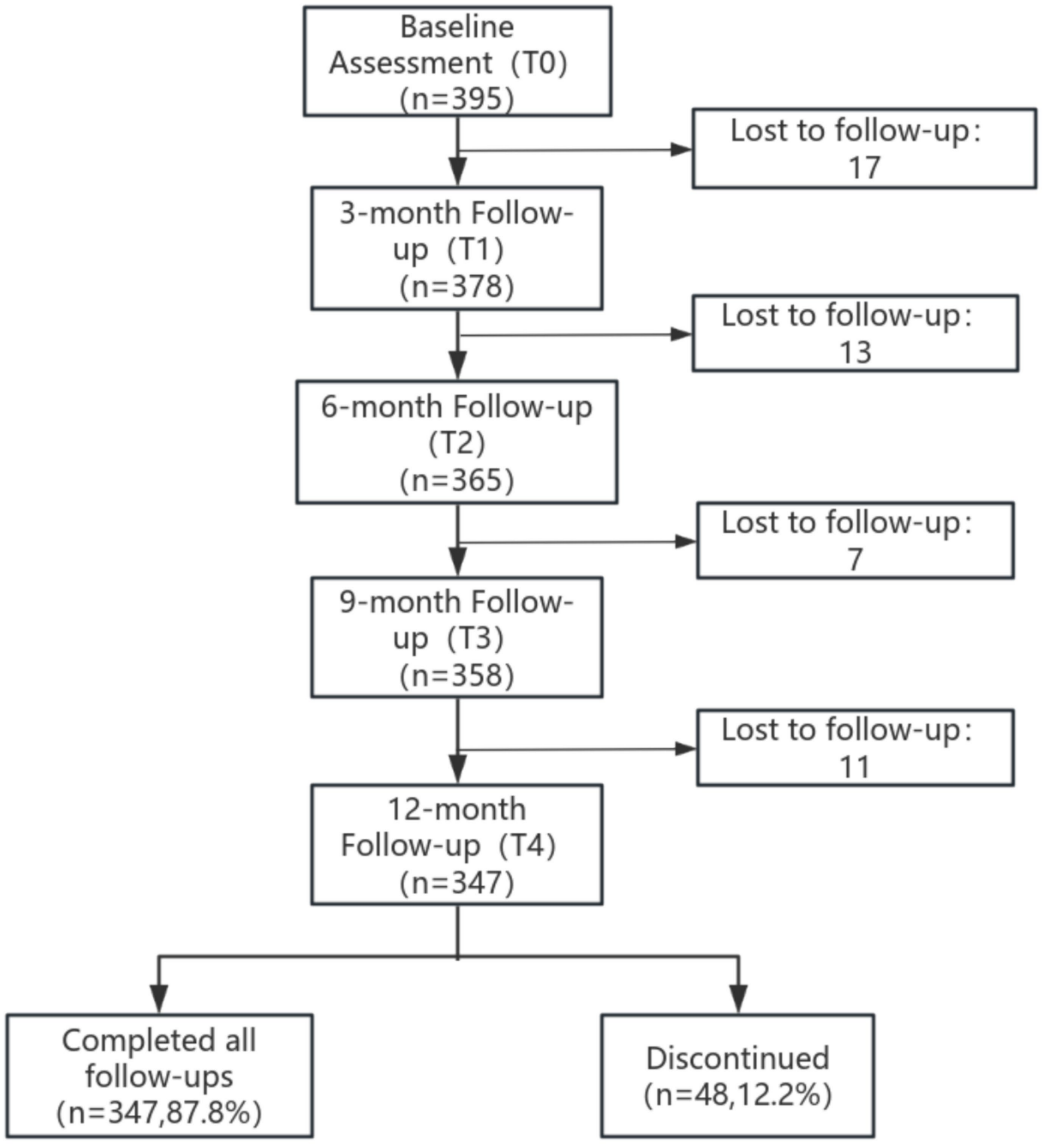
Participant enrollment and follow-up flowchart.

A total of 395 older adults with COPD were enrolled in this study, of whom 347 (87.8%) completed all five follow-ups. There were no significant differences in key baseline characteristics (age, sex, frailty, and lung function; *p* > 0.05) between completers and those lost to follow-up, indicating minimal bias from attrition. The participant flowchart is shown in Figure 1, and the complete baseline characteristics of the full sample are provided in Supplementary Table S1.

**Figure 2:**
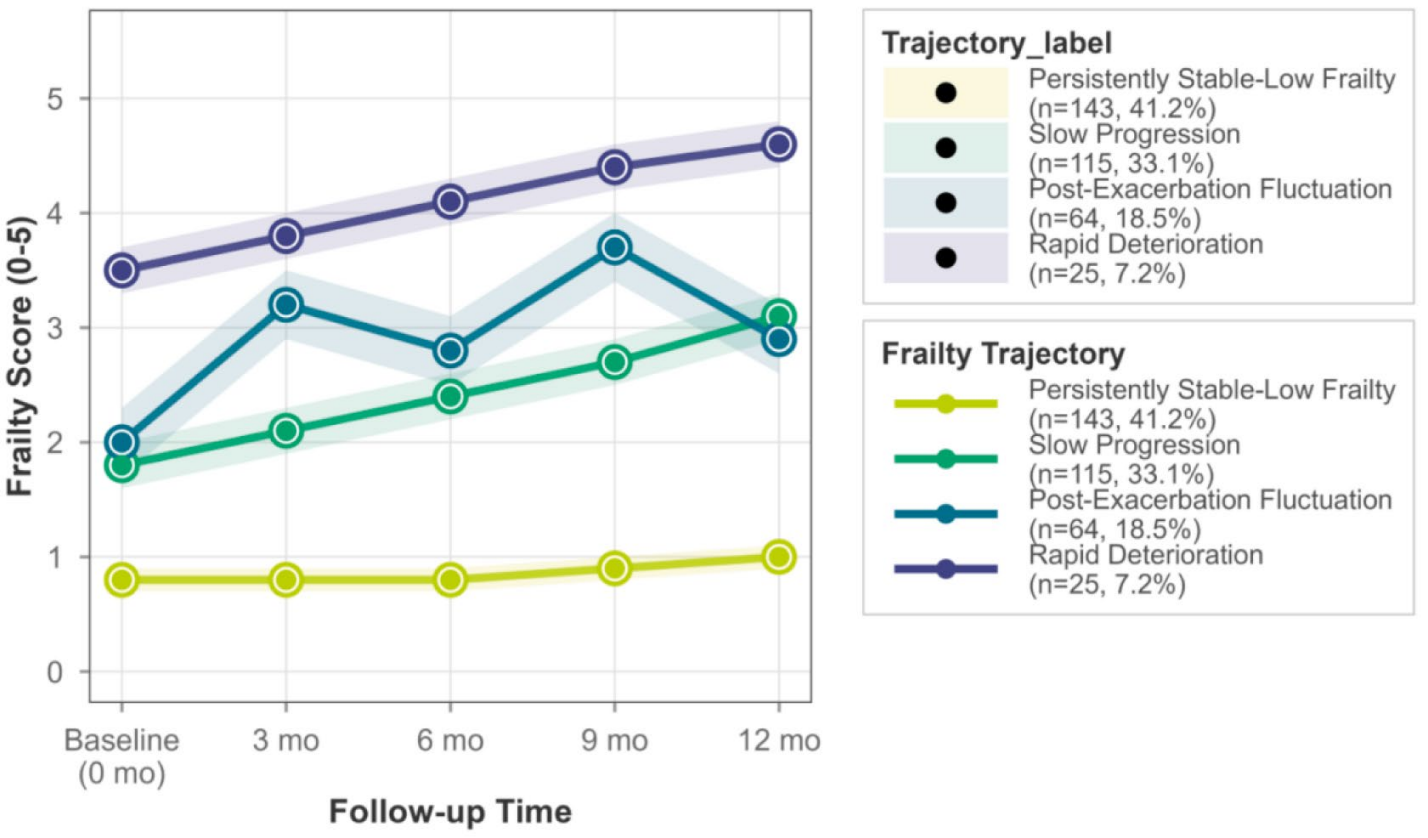
Four distinct frailty trajectories in older adults with COPD. This figure illustrates the longitudinal progression of frailty across four heterogeneous trajectories identified in 347 older adults with COPD during a 12-month follow-up period. The *x*-axis represents the follow-up time (baseline [0 months] to 12 months), and the *y*-axis represents the frailty score (range 0–5, with higher scores indicating greater frailty).

Using latent class growth analysis (R package lcmm) based on frailty scores at five time points, four heterogeneous trajectories were identified (Table 1). The 4-class model was determined to be optimal based on superior statistical fit indices (lower AIC, BIC, and aBIC; entropy > 0.8; significant LMR-LRT and BLRT) and clear clinical interpretability. These four trajectories (Figure 2) were characterized as follows: (I) Persistently Stable-Low Frailty (*n* = 143, 41.2%), maintaining consistently low scores (0.5–1.0 points); (II) Slow Progression (*n* = 115, 33.1%), showing a slow linear increase (from 1.8 ± 0.7 to 3.1 ± 0.9 points); (III) Post-Exacerbation Fluctuation (*n* = 64, 18.5%), exhibiting a unique pattern of “stepwise increase with partial recovery,” with score surges highly coinciding with the timing of moderate-to-severe acute exacerbation events; and (IV) Rapid Deterioration (*n* = 25, 7.2%), starting with the highest baseline score and demonstrating a sharp linear decline.

**Table 1:** Comparison of goodness-of-fit indices for frailty trajectory growth mixture models.

Number of classes	AIC	BIC	aBIC	Entropy	LMR-LRT (*p*-value)	BLRT (*p*-value)	Class probability (%)
1-class	4123.56	4156.78	4130.12	_	_	_	100
2-class	3987.23	4035.10	3997.89	0.821	0.012	<0.001	67.6/32.4
3-class	3901.45	3963.97	3916.21	0.835	0.043	<0.001	51.9/33.1/15.0
4-class	3845.12	3922.29	3863.98	0.851	0.030	<0.001	41.2/33.1/18.5/7.2
5-class	3832.88	3924.70	3855.84	0.849	0.127	0.102	40.3/30.3/15.3/8.1/6.1

AIC, Akaike Information Criterion; BIC, Bayesian Information Criterion; aBIC, sample-size adjusted BIC; LMR-LRT, Lo–Mendell–Rubin Adjusted Likelihood Ratio Test; BLRT, Bootstrap Likelihood Ratio Test.

To explore potential factors driving the heterogeneity in frailty trajectories, this study compared differences in baseline demographic characteristics, clinical features, functional status, and psychosocial indicators among the four trajectory groups. Results from univariate analysis (see Table 2) showed statistically significant differences (*p* < 0.05) in all characteristics among the four groups, except for sex, marital status, and living alone.

**Table 2:** Comparison of baseline characteristics among patients in different frailty trajectory groups.

Characteristic	Stable-low frailty (*n* = 143)	Slow progression (*n* = 115)	Post-exacerbation fluctuation (*n* = 64)	Rapid deterioration (*n* = 25)	Statistic (*F*/*χ*^2^/*H* value)	*p*-value
Demographics						
Age (years), M ± SD	70.1 ± 5.2	73.5 ± 6.8	74.8 ± 7.1	77.2 ± 6.5	*F* = 15.32	<0.001
Male, *n* (%)	90 (62.9%)	78 (67.8%)	42 (65.6%)	17 (68.0%)	*χ*^2^ = 0.85	0.837
Married, *n* (%)	110 (76.9%)	80 (69.6%)	44 (68.8%)	17 (68.0%)	*χ*^2^ = 2.95	0.399
Living Alone, *n* (%)	18 (12.6%)	21 (18.3%)	13 (20.3%)	6 (24.0%)	*χ*^2^ = 3.76	0.288
Years of Education, *M* ± SD	9.5 ± 4.2	8.1 ± 3.9	7.8 ± 4.1	7.2 ± 3.8	*H* = 12.45	0.006
Smoking (Pack-years), *M* ± SD	30.5 ± 15.8	35.2 ± 18.3	40.1 ± 20.5	45.8 ± 22.1	*H* = 25.67	<0.001
Clinical features						
FEV₁% predicted, *M* ± SD	63.5 ± 13.8	57.2 ± 14.1	54.8 ± 15.9	48.3 ± 16.2	*F* = 13.47	<0.001
GOLD Grade, *n* (%)					*χ*^2^ = 28.91	<0.001
Grade 1–2	98 (68.5%)	57 (49.6%)	29 (45.3%)	6 (24.0%)		
Grade 3–4	45 (31.5%)	58 (50.4%)	35 (54.7%)	19 (76.0%)		
Acute Exacerbations/year, *M* ± SD	0.8 ± 0.9	1.5 ± 1.1	2.4 ± 1.3	3.1 ± 1.5	*H* = 102.34	<0.001
No. of Comorbidities, *M* ± SD	2.1 ± 1.2	2.5 ± 1.3	3.0 ± 1.5	3.4 ± 1.3	*H* = 25.89	<0.001
Disease burden and symptoms						
CAT Total Score (0–40), M ± SD	16.5 ± 4.1	21.8 ± 5.3	23.4 ± 4.9	28.5 ± 5.7	*F* = 45.67	<0.001
mMRC Grade, *n* (%)					*χ*^2^ = 55.24	<0.001
Grade 0–1	92 (64.3%)	37 (32.2%)	16 (25.0%)	2 (8.0%)		
Grade ≥2	51 (35.7%)	78 (67.8%)	48 (75.0%)	23 (92.0%)		
Frailty and physical function						
Baseline Frailty Score (0–5), *M* ± SD	0.8 ± 0.4	1.8 ± 0.7	2.0 ± 0.8	3.5 ± 1.0	*H* = 210.15	<0.001
Unintentional Weight Loss, *n* (%)	8 (5.6%)	18 (15.7%)	15 (23.4%)	10 (40.0%)	*χ*^2^ = 32.15	<0.001
Grip Strength (kg), *M* ± SD	28.5 ± 5.8	25.1 ± 6.2	23.4 ± 5.9	20.7 ± 6.5	*F* = 18.92	<0.001
Exhaustion, *n* (%)	22 (15.4%)	35 (30.4%)	28 (43.8%)	18 (72.0%)	*χ*^2^ = 48.90	<0.001
Gait Speed (m/s), *M* ± SD	1.0 ± 0.2	0.8 ± 0.2	0.7 ± 0.2	0.6 ± 0.1	*F* = 35.41	<0.001
Low Physical Activity, *n* (%)	15 (10.5%)	30 (26.1%)	25 (39.1%)	16 (64.0%)	*χ*^2^ = 45.22	<0.001
Social integration (baseline)						
IADL Total Score (8–32), M ± SD	10.5 ± 2.5	12.8 ± 3.2	14.5 ± 4.1	18.2 ± 4.5	*H* = 85.22	<0.001
LSNS-6 Total Score (0–30), *M* ± SD	19.8 ± 5.1	16.0 ± 5.4	13.5 ± 4.9	9.8 ± 4.2	*F* = 35.60	<0.001
Social Isolation Risk (LSNS-6 < 12), *n* (%)	15 (10.5%)	28 (24.3%)	22 (34.4%)	11 (44.0%)	*χ*^2^ = 24.53	<0.001
UCLA Loneliness Total Score (20–80), *M* ± SD	35.5 ± 8.2	41.2 ± 9.5	46.8 ± 10.1	55.3 ± 11.8	*H* = 78.91	<0.001

*M* ± SD, Mean ± Standard Deviation; CAT, COPD Assessment Test; mMRC, modified Medical Research Council Dyspnea Scale; FEV₁, Forced Expiratory Volume in 1 s; IADL, Instrumental Activities of Daily Living; LSNS-6, 6-item Lubben Social Network Scale. For continuous variables, one-way ANOVA (reporting *F* value) was used if data were normally distributed and variances were homogeneous; otherwise, the Kruskal–Wallis *H* test (reporting *H* value) was used. Categorical variables were analyzed using the chi-square test (reporting *χ*^2^ value). *p* < 0.05 was considered statistically significant.

### 3.2 Comparison of social integration outcomes among frailty trajectory groups

To examine the association between frailty development trajectories and long-term social integration outcomes, this study compared the levels of social integration at baseline (T0) and 12 months (T4) across the four trajectory groups, and analyzed the change values (*Δ*), as shown in Table 3. One-way analysis of variance results indicated statistically significant differences among the different frailty trajectory groups in both the T4 scores and the change values from baseline to T4 (Δ) across the three social integration dimensions (all *p* < 0.001). The social integration outcomes exhibited a clear gradient of worsening, progressing from the “Persistently Stable-Low Frailty” group to the “Rapid Deterioration” group (Table 3). In terms of functional integration (IADL), the between-group differences were significant. The “Rapid Deterioration” group had the most severe IADL dysfunction at T4, with a mean score of 21.4 points, and their function significantly worsened over the year (*Δ* = +3.2 points). In contrast, the function of the “Persistently Stable-Low Frailty” group remained stable.

**Table 3:** Comparison of social integration dimension scores at 12 months among different frailty trajectory groups.

Social integration dimension	Persistently stable-low frailty (*n* = 143)	Slow progression (*n* = 115)	Post-exacerbation fluctuation (*n* = 64)	Rapid deterioration (*n* = 25)	*F*/*χ*^2^ value	*P*-value	*Post hoc* comparison (LSD)
Functional integration (IADL total score)							
Baseline (T0), *M* ± SD	10.5 ± 2.5	12.8 ± 3.2	14.5 ± 4.1	18.2 ± 4.5	85.22	<0.001	1 < 2 < 3 < 4
12-month (T4), *M* ± SD	10.2 ± 2.3	13.5 ± 3.1	16.8 ± 3.9	21.4 ± 4.2	185.63	<0.001	1 < 2 < 3 < 4
Δ (T4-T0), *M* ± SD	−0.3 ± 1.5	0.7 ± 1.8	2.3 ± 2.1	3.2 ± 2.8	45.20	<0.001	1 < 2 < 3 < 4
Structural integration (LSNS-6 total score)							
Baseline (T0), *M* ± SD	19.8 ± 5.1	16.0 ± 5.4	13.5 ± 4.9	9.8 ± 4.2	35.6	<0.001	1 > 2 > 3 > 4
12-month (T4), *M* ± SD	20.5 ± 4.8	16.1 ± 5.2	12.4 ± 4.7	8.3 ± 3.9	95.47	<0.001	1 > 2 > 3 > 4
Δ (T4-T0), *M* ± SD	0.7 ± 3.2	0.1 ± 3.5	−1.1 ± 3.8	−1.5 ± 4.1	5.98	<0.001	1 > 2,3,4; 2,3 > 4
Social Isolation (T4, LSNS-6 < 12), *n* (%)	9 (6.3%)	31 (27.0%)	30 (46.9%)	18 (72.0%)	*χ*^2^ = 86.32	<0.001	1 < 2 < 3 < 4
Subjective integration experience (UCLA loneliness)							
Baseline (T0), *M* ± SD	35.5 ± 8.2	41.2 ± 9.5	46.8 ± 10.1	55.3 ± 11.8	78.91	<0.001	1 < 2 < 3 < 4
12-month (T4), *M* ± SD	35.2 ± 7.5	42.8 ± 8.3	49.5 ± 9.1	58.6 ± 10.2	103.24	<0.001	1 < 2 < 3 < 4
Δ (T4-T0), *M* ± SD	−0.3 ± 6.1	1.6 ± 6.8	2.7 ± 7.5	3.3 ± 8.9	4.62	0.004	1 < 2 < 3 < 4

IADL, Instrumental Activities of Daily Living scale, higher scores indicate greater functional impairment; LSNS-6, 6-item Lubben Social Network Scale, higher scores indicate better social networks; UCLA, UCLA Loneliness Scale (Version 3), higher scores indicate stronger loneliness. M ± SD, Mean ± Standard Deviation. Continuous variables were analyzed using one-way ANOVA, categorical variables using the chi-square test. In the *post hoc* comparison column, numbers 1, 2, 3, 4 represent the “Persistently Stable-Low Frailty”, “Slow Progression”, “Post-Exacerbation Fluctuation”, and “Rapid Deterioration” groups, respectively. “<” or “>” indicates a statistically significant difference between adjacent groups.

Regarding structural integration (LSNS-6), the size of the social network showed an inverse gradient with the frailty trajectory. The LSNS-6 total score (T4) decreased significantly from 20.5 points in the “Persistently Stable-Low Frailty” group to 8.3 points in the “Rapid Deterioration” group. The latter group experienced an average shrinkage of 1.5 points in their social network over the year (*Δ* = -1.5 points). It is noteworthy that 72.0% of the patients in the “Rapid Deterioration” group were in a state of social isolation (LSNS-6 < 12 points) at T4.

For subjective integration experience (UCLA Loneliness), the loneliness scores increased in a stepwise manner as the frailty trajectories worsened. The UCLA total score (T4) rose from 35.2 points in the “Persistently Stable-Low Frailty” group to 58.6 points in the “Rapid Deterioration” group. The latter group experienced an average increase in loneliness of 3.3 points over the year (*Δ* = +3.3 points).

### 3.3 Association between frailty trajectories and social integration outcomes

To clarify the independent association of frailty trajectories with long-term social integration outcomes, hierarchical multiple linear regression analysis was employed, evaluating the strength of association after controlling for relevant variables. As shown in Table 4, frailty trajectories had a significant and gradient independent association with social integration outcomes at 12 months, and this association remained stable across different adjustment models.

**Table 4:** Association between frailty trajectories and 12-month social integration outcomes: hierarchical multiple linear regression analysis.

Outcome and predictor	Model 1: *β* (95% CI)	Model 2: *β* (95% CI)	Model 3: *β* (95% CI)
IADL total score (8–32)			
Slow Progression	2.92 (1.41, 4.43)^***^	2.88 (1.37, 4.39)^***^	2.85 (1.32, 4.38)^***^
Post-exacerbation fluctuation	5.73 (3.84, 7.62)^***^	5.69 (3.80, 7.58)^***^	5.67 (3.78, 7.56)^***^
Rapid deterioration	9.48 (7.21, 11.75)^***^	9.44 (7.17, 11.71)^***^	9.42 (7.15, 11.69)^***^
LSNS-6 total score (0–30)			
Slow progression	−3.15 (−4.88, −1.42)^***^	−3.14 (−4.87, −1.41)^***^	−3.12 (−4.85, −1.39)^***^
Post-exacerbation fluctuation	−6.26 (−8.48, −4.04)^***^	−6.25 (−8.47, −4.03)^***^	−6.23 (−8.45, −4.01)^***^
Rapid deterioration	−10.21 (−12.84, −7.58)^***^	−10.18 (−12.81, −7.55)^***^	−10.15 (−12.78, −7.52)^***^
UCLA loneliness score (20–80)			
Slow progression	7.60 (4.89, 10.31)^***^	7.58 (4.87, 10.29)^***^	7.56 (4.85, 10.27)^***^
Post-exacerbation fluctuation	12.41 (9.27, 15.55)^***^	12.39 (9.25, 15.53)^***^	12.37 (9.23, 15.51)^***^
Rapid deterioration	18.28 (13.49, 23.07)^***^	18.26 (13.47, 23.05)^***^	18.24 (13.45, 23.03)^***^

(1) Reference Group: The “Persistently Stable-Low Frailty” trajectory group served as the reference in all models. (2) Model Specification: Model 1: Adjusted for the corresponding baseline social integration score (baseline IADL, LSNS-6, or UCLA Loneliness). Model 2: Adjusted for variables in Model 1 plus demographic variables (age, sex, and years of education). Model 3: Adjusted for variables in Model 2 plus clinical variables (FEV₁% predicted, annual frequency of acute exacerbations). This is the final, fully adjusted model.

(3) Interpretation:β: Unstandardized regression coefficient. For IADL and UCLA models, *β* > 0 indicates that the trajectory is associated with worse function/greater loneliness compared to the reference group. For the LSNS-6 model, *β* < 0 indicates association with a smaller social network. 95% CI: 95% confidence interval. An interval not containing zero indicates statistical significance. ***denotes *p* < 0.001.

Complete Results: The full regression coefficients, confidence intervals, and statistics for all control variables (age, sex, education, FEV₁%, acute exacerbations, baseline level) in each model are available in Supplementary Table S2.

Regarding functional integration (IADL), compared to the “Persistently Stable-Low Frailty” group, the “Slow Progression,” “Post-Exacerbation Fluctuation,” and “Rapid Deterioration” trajectory groups all showed significantly higher IADL dysfunction scores (*β* = 2.85, 5.67, 9.42, respectively, in Model 3; all *p* < 0.001). For structural integration (LSNS-6), worse frailty trajectories were significantly associated with smaller social network sizes (*β* = −3.12, −6.23, −10.15, respectively, in Model 3; all *p* < 0.001). Regarding subjective integration experience (UCLA Loneliness), frailty trajectories were also significantly associated with greater loneliness (*β* = 7.56, 12.37, 18.24, respectively, in Model 3; all *p* < 0.001).

From Model 1 (adjusted only for baseline level) to Model 2 (further including demographic variables) and finally to the fully adjusted Model 3, the corresponding regression coefficients for each trajectory changed minimally, indicating good robustness of the association between frailty trajectories and social integration outcomes. Complete regression results for the control variables are available in Supplementary Table S2.

### 3.4 Exploratory and sensitivity analyses

#### 3.4.1 Sensitivity analysis of trajectory classification

To evaluate the clinical feasibility of using a shorter follow-up period for early identification of frailty trajectories, we re-ran the Growth Mixture Model (GMM) using data from the first 6 months (baseline, 3 months, 6 months). The GMM based on 6-month data also identified 4 potential classes, with a patient distribution highly similar to the original model based on 12-month data. The agreement between the trajectory classifications of the two models was good (Cohen’s *κ* = 0.72, 95% CI: 0.65–0.79, *p* < 0.001). Specifically, 89.6% (311/347) of the patients were classified into the same trajectory group by both the 6-month and 12-month models. Agreement was highest for the “Persistently Stable-Low Frailty” group (94.4%) and relatively lowest, though still acceptable, for the “Rapid Deterioration” group (80.0%) (Table 5). These results indicate that patients’ frailty change patterns within the first 6 months can effectively reflect their longer-term trajectories, supporting the potential value of using a shorter observation period (e.g., 6 months) for early risk warning in clinical practice.

**Table 5:** Agreement analysis of frailty trajectory classification based on 6-month vs. 12-month data.

12-month trajectory classification	No. of patients (n)	No. of Patients with agreement in 6-month classification, *n* (%)	Agreement (Cohen’s *κ*, 95% CI)
Persistently stable-low frailty	143	135 (94.4%)	0.72 (0.65–0.79)
Slow progression	115	100 (87.0%)	
Post-exacerbation fluctuation	64	56 (87.5%)	
Rapid deterioration	25	20 (80.0%)	
Total	347	311 (89.6%)	

#### 3.4.2 Interaction analysis

To explore potential effect modification, we examined both multiplicative and additive interaction between frailty trajectories and two key clinical characteristics (lung function, acute exacerbation frequency) on social integration outcomes. Multiplicative interaction was assessed by introducing product terms (frailty trajectory × clinical characteristic) into the final multiple linear regression models (Model 3). Additive interaction, which is of particular public health relevance for identifying high-risk subgroups, was evaluated using the relative excess risk due to interaction (RERI), the attributable proportion due to interaction (AP), and the synergy index (S), based on the regression coefficients from the fully adjusted models. Lung function was dichotomized into “Better” (FEV₁% predicted ≥ median, *n* = 176) and “Poorer” (FEV₁% predicted < median, *n* = 171) groups. Acute exacerbation frequency was dichotomized into “Low frequency” (annual exacerbations ≤ median, *n* = 221) and “High frequency” (annual exacerbations > median, *n* = 126) groups. As shown in Table 6, the multiplicative interaction between frailty trajectory and either clinical characteristic was not statistically significant in any of the three social integration outcome models (all interaction *p* > 0.10). Similarly, the additive interaction measures (RERI, AP, S) for all tested combinations had confidence intervals spanning the null value (1 for RERI and S, 0 for AP), indicating no statistically significant additive interaction (Table 6).

**Table 6:** Interaction analysis between frailty trajectory and key clinical characteristics.

Interaction and metric	IADL model	LSNS-6 model	UCLA loneliness model
Frailty trajectory × Lung function group			
Multiplicative interaction			
*β* (95% CI)	0.15 (−0.21, 0.51)	−0.18 (−0.58, 0.22)	0.31 (−0.25, 0.87)
*p*-value	0.412	0.376	0.279
Additive interaction			
RERI (95% CI)	0.85 (−1.12, 2.82)	−0.62 (−2.45, 1.21)	1.08 (−2.15, 4.31)
AP (95% CI)	0.09 (−0.15, 0.33)	−0.10 (−0.42, 0.22)	0.06 (−0.13, 0.25)
S (95% CI)	1.10 (0.85, 1.42)	0.90 (0.66, 1.23)	1.06 (0.87, 1.29)
Frailty Trajectory × Acute Exacerbation Frequency Group			
Multiplicative interaction			
*β* (95% CI)	0.09 (−0.28, 0.46)	−0.12 (−0.53, 0.29)	0.22 (−0.35, 0.79)
*p*-value	0.623	0.569	0.448
Additive interaction			
RERI (95% CI)	0.45 (−1.55, 2.45)	−0.41 (−2.28, 1.46)	0.78 (−2.50, 4.06)
AP (95% CI)	0.05 (−0.20, 0.30)	−0.07 (−0.40, 0.26)	0.04 (−0.15, 0.23)
S (95% CI)	1.05 (0.80, 1.38)	0.93 (0.68, 1.28)	1.04 (0.85, 1.28)

## 4 Discussion

This 12-month prospective longitudinal cohort study systematically investigated the heterogeneous trajectories of frailty development and their association with long-term social integration outcomes in older adults with chronic obstructive pulmonary disease (COPD). The main findings suggest that the frailty progression in this population is not homogeneous but can be clearly categorized into four subgroups with different clinical prognostic implications. More importantly, these trajectory classes are strong indicators for predicting patients’ instrumental activities of daily living, social network size, and subjective loneliness 12 months later. These findings not only methodologically advance the understanding of the dynamic process of COPD-related frailty but also provide key theoretical and empirical evidence for constructing a risk-stratified management system and implementing individualized nursing interventions in clinical practice.

The four frailty trajectories identified in this study likely result from the interplay of multiple factors. A preliminary analysis of the potential causes of each trajectory can be conducted based on the study’s baseline data and clinical observations. The formation of the “Persistently Stable-Low Frailty” trajectory (41.2%) may primarily benefit from relatively favorable baseline physiological conditions and a mild disease course. This group had the youngest average age (70.1 years), better lung function, the fewest acute exacerbations in the past year, and the lowest proportion in a frail state at baseline. This suggests that better initial physiological reserve and low disease activity may together form a foundation for resisting rapid functional decline (24), enabling them to maintain a stable frailty status during the 12-month follow-up. The “Slow Progression” trajectory (33.1%) may reflect the continuous attrition under the combined effects of COPD as a chronic progressive disease and aging. This group already had a moderate level of frailty at baseline, accompanied by a more frequent history of acute exacerbations and higher CAT symptom scores. This persistent disease burden may gradually but slowly erode their physiological functional reserve, leading to a nearly linear increase in frailty scores (25). This aligns with the natural history of COPD as a progressive disease, manifesting in this subgroup as a relatively “gradual” decline pattern. The unique shape of the “Post-Exacerbation Fluctuation” trajectory (18.5%) directly highlights the central role of acute exacerbation events as key drivers and shapers. This group had the highest annual frequency of acute exacerbations. Each moderate-to-severe exacerbation event is accompanied by significant systemic inflammatory responses, activity limitation, and potential complications, which may lead to a precipitous drop in core frailty indicators like muscle strength and exercise tolerance, manifesting as a “leap” in frailty scores (26, 27). Although scores may partially recover after treatment post-acute phase, each episode may cause some irreversible functional loss, preventing a full return to pre-event levels, thus macroscopically forming a “leap-partial recovery-further leap” stepwise downward trajectory. This suggests that for these patients, the focus of clinical intervention should be preemptive, i.e., making every effort to prevent the occurrence of acute exacerbations. Finally, the “Rapid Deterioration” trajectory (7.2%) may be the severe consequence of the superposition and combined action of multiple high-risk baseline factors. This group exhibited comprehensive extreme disadvantages at baseline: oldest age, poorest lung function, most frequent acute exacerbations, highest number of comorbidities, and their objective functional indicators like grip strength and gait speed were at the lowest level from the start. This extremely fragile physiological foundation provides a very low buffer capacity against any internal or external stressors, including disease progression itself, treatment side effects, or incidental infections. Even minor stressors may lead to severe functional decompensation, resulting in rapid deterioration of frailty status in a short time (28).

A core finding of this study is the indication that different frailty development trajectories at baseline are significantly associated with patients’ social integration levels at 12 months, and this association shows a clinically meaningful gradient. This not only provides a basis for understanding the social significance of frailty as a comprehensive health indicator but also offers a reference for exploring risk-stratified management based on trajectories. From the perspective of “functional integration,” frailty trajectories are important factors associated with changes in patients’ instrumental activities of daily living. The data from this study show significant gradient differences in IADL dysfunction levels among the four groups at 12 months. The “Rapid Deterioration” group had a relatively high average IADL score, approaching the scale-defined range of “severe dysfunction,” suggesting that patients in this group may generally require more assistance with complex life tasks such as shopping and transportation. After controlling for age, lung function, and particularly baseline IADL levels, multiple linear regression analysis showed that the “Rapid Deterioration” trajectory was independently and significantly associated with IADL worsening. This may indicate that the rapid progression of frailty is an important factor for understanding the decline in patients’ social role functioning.

Secondly, at the level of “structural integration,” frailty trajectories also show associations with changes in objective social network resources. Social network size exhibits a gradient trend opposite to that of the frailty trajectories. The “Rapid Deterioration” group had a relatively low average LSNS-6 score at 12 months, well below the commonly used risk threshold for social isolation, and a high proportion of patients in this group were in a state of social isolation. Regression analysis suggests that, even after controlling for baseline social network status, the “Rapid Deterioration” trajectory remains independently associated with a decline in LSNS-6 scores. A possible explanation for this result is that the rapid progression of frailty may, on one hand, physically hinder patients from visiting friends or participating in community activities due to severe physical activity limitations (29). On the other hand, it may also lead patients to proactively reduce social engagement for psychological reasons, such as feeling like a “burden” or due to changes in self-image (30). A history of frequent acute exacerbations also showed an independent association in the model, suggesting that acute exacerbation events may need to be considered as a factor when attending to changes in social connectedness.

Regarding “subjective integration experience,” different frailty trajectories are associated with patients’ perceived loneliness. Subjective loneliness scores showed an increasing trend from the “Stable” group to the “Rapid Deterioration” group, with the latter’s average score reaching the range of severe loneliness. This suggests that patients experiencing rapid frailty progression may simultaneously face challenges in both social situations and emotional experience. The association between the “Rapid Deterioration” trajectory and loneliness in the multivariate regression analysis also indicates that the rapid decline in physiological function and the worsening of psychosocial experience may coexist in these patients. The interpretation of these associations must be grounded in the design limitations of an observational study. This study reveals significant and independent statistical associations but cannot establish strict causality. The observed relationships may be due to frailty directly leading to a decline in social integration, or there may be common unmeasured factors (such as subclinical inflammation, specific genetic predispositions) driving both simultaneously, or bidirectional influences may exist. Therefore, this study avoids using terms like “strong prediction” that might imply definitive causality, instead emphasizing that heterogeneous frailty trajectories are important associated indicators and risk stratification markers for social integration outcomes.

At the theoretical level, the fear-avoidance model provides a preliminary framework for understanding the vicious cycle of “dyspnea → activity limitation → social withdrawal.” The association patterns observed in this study are consistent with this model and can be further integrated into broader theories. For example, the Disablement Process Model emphasizes the progression from impairment to activity limitation to participation restriction. In this study, frailty, as a comprehensive manifestation of multi-system impairment, may be a key hub accelerating this process and leading to a decline in social integration. Furthermore, Socioemotional Selectivity Theory posits that individuals adjust their social goals based on their perceived future time. The rapid progression of frailty may significantly shorten a patient’s perceived “future time,” thereby prompting them to actively or passively reduce their social circle, prioritizing disengagement from non-core social roles and networks. These theoretical perspectives collectively suggest that paying attention to the dynamic trajectory of frailty is of great significance for understanding the evolution of patients’ social functioning.

Based on this, the findings of this study have implications for clinical practice, particularly for promoting individualized care. First, it supports the integration of dynamic frailty assessment into the routine management of COPD patients, focusing not only on their status but also on their change trends, to facilitate the early identification of patients with different risk trajectory characteristics. Secondly, the study inspires the conceptualization of differentiated nursing support pathways based on trajectory features: for the “Stable-Low Frailty” group, care should focus on maintenance and health promotion; for the “Slow Progression” group, proactive intervention is needed to delay functional decline; for the “Post-Exacerbation Fluctuation” group, the key lies in optimizing transitional care during acute exacerbations and functional recovery post-event; for the “Rapid Deterioration” group, it is necessary to initiate multidisciplinary integrated care, managing complex medical issues while striving to maintain their social connectedness and quality of life. Future research needs to validate the stability of these trajectories in larger, more culturally diverse cohorts and design interventional studies to empirically test the effectiveness of such stratified care pathways.

This study has several limitations that require careful consideration. First, on the methodological level, although Growth Mixture Modeling provides a powerful tool for identifying potential heterogeneous trajectories, its application has inherent limitations. Determining the optimal number of classes combines statistical indices with researcher judgment of clinical significance, involving a degree of subjectivity. Although we applied strict fit criteria (entropy > 0.8, significant LMR test), the smallest trajectory, “Rapid Deterioration” (7.2%), is statistically relatively unstable, and its reproducibility in different samples or with different modeling strategies requires verification in future studies. Furthermore, the model assumes data are missing at random; if the missing data mechanism deviates from this, it may affect the trajectory estimation to some extent. Second, regarding the study sample and context, the single-center design means the sample may not be fully representative in terms of disease severity, treatment accessibility, and socioeconomic characteristics, limiting the generalizability of the findings to community populations, patients in other levels of healthcare institutions, or populations within other healthcare systems. More importantly, this study is rooted in the specific socio-cultural context of China. China’s family-centered support system, traditions of intergenerational co-residence, and emphasis on “filial piety” profoundly shape the structure and function of older adults’ social networks. This may mean that the measurement connotation of social integration (especially structural integration), its baseline level, and its relationship pattern with frailty differ significantly from Western societies that emphasize individual independence and the nuclear family. Therefore, the cultural universality of this study’s conclusions needs to be tested through cross-cultural comparative research. Third, concerning causal inference, despite using a prospective design and statistical controls to reduce confounding, as an observational study, it cannot completely rule out the influence of unmeasured confounding factors (e.g., specific genetic susceptibility, early life environment, or unevaluated psychosocial resources) on the observed “trajectory-outcome” associations. Therefore, what we report are primarily statistically independent associations, not definitive causal relationships.

Finally, regarding the temporal dimension, the 12-month follow-up period is sufficient to observe significant short-term changes. However, some aspects of social integration (e.g., the dissolution of deep friendship networks, the complete transformation of social identity) may be a more prolonged, non-linear process. This study could not capture these long-term evolutionary trajectories, nor could it assess the potential bidirectional dynamic relationship between frailty trajectories and social integration. Based on the above, future research could delve deeper in the following directions: First, validate the trajectories identified in this study in larger populations and over longer follow-up periods, and explore their biomarkers. Second, develop and validate prediction models based on preliminary trajectory classification for early clinical application. Third, and most practical, is to design and implement randomized controlled nursing intervention trials targeting different frailty trajectory subgroups to empirically test the cost-effectiveness of “stratified precision care pathways” in improving both physiological and social outcomes for patients.

## 5 Conclusion

This study, from a prospective dynamic perspective, reveals that frailty development in older adults with COPD follows heterogeneous trajectories, which can be systematically summarized into four categories with different clinical and social prognoses. These trajectory categories are strong independent predictors of long-term social integration outcomes, showing a clear gradient association. In the clinical management of COPD, especially nursing practice, dynamic assessment of frailty trajectories should be considered a core component of evaluation. This should drive a fundamental shift in the nursing model from universal approaches toward risk-stratified, precision intervention. By early identification of high-risk trajectories and implementing matching multidimensional nursing support, it may be possible to effectively delay frailty progression, block its transmission path to social functional loss, and ultimately enhance the overall health and quality of life for this growing population of older adults with chronic disease.

## Data Availability

The original contributions presented in the study are included in the article/Supplementary material, further inquiries can be directed to the corresponding author.
