# Supplementary material for: Heterogeneous frailty trajectories and their differential impact on social integration in older adults with COPD: a prospective longitudinal study
Source: Front Public Health. 2026 May 8;14:1824141. doi: 10.3389/fpubh.2026.1824141 (PMC13194501; doi:10.3389/fpubh.2026.1824141)
Supplement: Supplementary file 1 [file Table_1.docx]

Table S1. Baseline Characteristics of Older Adults with COPD (N=347)

| Characteristic | Category/Value | Statistics |
| --- | --- | --- |
| Demographics |  |  |
| Age (years) | — | 72.5 ± 6.8 |
| Years of Education |  | 9.1 ± 4.0 |
| Sex, n (%) | Male | 227 (65.4%) |
|  | Female | 120 (34.6%) |
| Marital Status, n (%) | Married | 251 (72.3%) |
|  | Not Married | 96 (27.7%) |
| Living Arrangements, n (%) | Living Alone | 58 (16.7%) |
|  | Living with Non-Spouse | 45 (13.0%) |
|  | Living with Spouse/Partner | 244 (70.3%) |
| Clinical Features |  |  |
| COPD GOLD Grade, n (%) | Grade 1 | 28 (8.1%) |
|  | Grade 2 | 172 (49.6%) |
|  | Grade 3 | 119 (34.3%) |
|  | Grade 4 | 28 (8.1%) |
| FEV₁% predicted | — | 58.4 ± 15.2 |
| Acute Exacerbations (past year) | — | 1.8 ± 1.3 |
| Number of Comorbidities | — | 2.5 ± 1.4 |
| Frailty Status (Fried Phenotype), n (%) | Non-frail (0 criteria) | 125 (36.1%) |
|  | Pre-frail (1-2 criteria) | 157 (45.2%) |
|  | Frail (≥3 criteria) | 65 (18.7%) |
| Frailty Composite Score (0-5) | — | 1.5 ± 1.2 |
| Disease Burden & Symptoms |  |  |
| CAT Total Score (0-40) |  | 20.3 ± 6.7 |
| mMRC Dyspnea Scale (0-4) |  | 1.9 ± 0.8 |
| Social Integration (Baseline) |  |  |
| Social Isolation Risk (LSNS-6 <12), n (%) | Yes | 76 (21.9%) |
|  | No | 271 (78.1%) |
| LSNS-6 Total Score (0-30) |  | 16.8 ± 5.9 |
| IADL Total Score (8-32) |  | 12.5 ± 4.3 |
| UCLA Loneliness Total Score (20-80) |  | 40.1 ± 10.6 |

Note: Data are presented as mean ± standard deviation or n (%). Abbreviations: COPD, Chronic Obstructive Pulmonary Disease; GOLD, Global Initiative for Chronic Obstructive Lung Disease; FEV₁%, forced expiratory volume in one second percent predicted; CAT, COPD Assessment Test; mMRC, modified Medical Research Council; LSNS-6, 6-item Lubben Social Network Scale; IADL, Instrumental Activities of Daily Living; UCLA, University of California, Los Angeles Loneliness Scale (Version 3).
