# Supplementary material for: Heterogeneous frailty trajectories and their differential impact on social integration in older adults with COPD: a prospective longitudinal study
Source: Front Public Health. 2026 May 8;14:1824141. doi: 10.3389/fpubh.2026.1824141 (PMC13194501; doi:10.3389/fpubh.2026.1824141)
Supplement: Supplementary file 2 [file Table_2.docx]

Supplementary Table S2. Complete Results of Hierarchical Multiple Linear Regression Analysis of Frailty Trajectories on 12-Month Social Integration Outcomes (n=347)

| **Predictor** | **IADL Total Score (8-32)** | | | **LSNS-6 Total Score (0-30)** | | | **UCLA Loneliness (20-80)** | | |
| --- | --- | --- | --- | --- | --- | --- | --- | --- | --- |
|  | Model 1 | Model 2 | Model 3 | Model 1 | Model 2 | Model 3 | Model 1 | Model 2 | Model 3 |
|  | β (95% CI) | β (95% CI) | β (95% CI) | β (95% CI) | β (95% CI) | β (95% CI) | β (95% CI) | β (95% CI) | β (95% CI) |
| ****Frailty Trajectory (Ref: Persistently Stable-Low Frailty)**​** | | | |  |  |  |  |  |  |
| Slow Progression | 2.92 (1.41, 4.43) | 2.88 (1.37, 4.39) | 2.85 (1.32, 4.38) | -3.15 (-4.88, -1.42) | -3.14 (-4.87, -1.41) | -3.12 (-4.85, -1.39) | 7.60 (4.89, 10.31) | 7.58 (4.87, 10.29) | 7.56 (4.85, 10.27) |
| Post-Exacerbation Fluctuation | 5.73 (3.84, 7.62) | 5.69 (3.80, 7.58) | 5.67 (3.78, 7.56) | -6.26 (-8.48, -4.04) | -6.25 (-8.47, -4.03) | -6.23 (-8.45, -4.01) | 12.41 (9.27, 15.55) | 12.39 (9.25, 15.53) | 12.37 (9.23, 15.51) |
| Rapid Deterioration | 9.48 (7.21, 11.75) | 9.44 (7.17, 11.71) | 9.42 (7.15, 11.69) | -10.21 (-12.84, -7.58) | -10.18 (-12.81, -7.55) | -10.15 (-12.78, -7.52) | 18.28 (13.49, 23.07) | 18.26 (13.47, 23.05) | 18.24 (13.45, 23.03) |
| **Demographics** |  |  |  |  |  |  |  |  |  |
| Age (per year) | — | 0.13 (0.06, 0.20) | 0.12 (0.05, 0.19) | — | -0.16 (-0.25, -0.07) | -0.15 (-0.24, -0.06) | — | 0.29 (0.12, 0.46) | 0.28 (0.11, 0.45) |
| Sex (Male vs. Female) | — | -0.33 (-1.10, 0.44) | -0.35 (-1.12, 0.42) | — | 0.43 (-0.44, 1.30) | 0.42 (-0.45, 1.29) | — | -0.76 (-2.13, 0.61) | -0.78 (-2.15, 0.59) |
| Education (years) | — | -0.19 (-0.32, -0.06) | -0.18 (-0.31, -0.05) | — | 0.23 (0.09, 0.37) | 0.22 (0.08, 0.36) | — | -0.36 (-0.56, -0.16) | -0.35 (-0.55, -0.15) |
| **Clinical Variables** |  |  |  |  |  |  |  |  |  |
| FEV₁% predicted (per 1%) | — | — | -0.04 (-0.08, -0.001) | — | — | 0.05 (0.00, 0.10) | — | — | -0.08 (-0.15, -0.01) |
| Acute exacerbations/year (per event) | — | — | 0.68 (0.35, 1.01) | — | — | -0.75 (-1.12, -0.38) | — | — | 1.25 (0.72, 1.78) |
| **Baseline Level** |  |  |  |  |  |  |  |  |  |
| Baseline IADL (per point) | 0.47 (0.34, 0.60) | 0.46 (0.33, 0.59) | 0.45 (0.32, 0.58) | — |  | — | — | — | — |
| Baseline LSNS-6 (per point) | — | — | — | 0.40 (0.26, 0.54) |  | 0.38 (0.24, 0.52) | — | — | — |
| Baseline UCLA Loneliness (per point) | — | — | — | — |  | — | 0.33 (0.20, 0.46) | 0.32 (0.19, 0.45) | 0.31 (0.18, 0.44) |
| Model Statistics |  |  |  |  |  |  |  |  |  |
| Adjusted R² | 0.385 | 0.402 | 0.412 | 0.345 | 0.368 | 0.368 | 0.372 | 0.388 | 0.395 |
| F value | 18.45 | 14.23 | 15.78 | 15.67 | 12.94 | 12.94 | 17.82 | 13.45 | 14.65 |
| P value | ＜0.001 | ＜0.001 | ＜0.001 | ＜0.001 | ＜0.001 | <0.001 | <0.001 | ＜0.001 | ＜0.001 |

Note: β: unstandardized regression coefficient; CI: confidence interval. — indicates the variable was not included in the corresponding model.Model 1: Adjusted for the corresponding baseline social integration level (baseline IADL for IADL model, baseline LSNS-6 for LSNS-6 model, baseline UCLA Loneliness for UCLA model).Model 2: Adjusted for variables in Model 1 plus demographic variables (age, sex, years of education).Model 3: Adjusted for variables in Model 2 plus clinical variables (FEV₁% predicted, annual frequency of acute exacerbations).
